# Lipodystrophy and metabolic syndrome in Romanian HIV-infected adults

**DOI:** 10.1186/1758-2652-13-S4-P71

**Published:** 2010-11-08

**Authors:** M Arbune

**Affiliations:** 1 Infectious Diseases Hospital, HIV/AIDS, Galati, Romania

## Background

Lipoatrophy (LA), lipohypertrophy (LH) and mixed features of lipodystrophy (LD) are variable observed in human immunodeficiency virus (HIV)-infected patients. Dyslipidaemia is frequently reported as the overlap disorder of HIV related LD and metabolic syndrome (MS). Moreover, waist circumference could be a common measure of LH and MS criteria.

## Objective

To assess the connection between LD and MS on HIV adult patients.

## Methods

76 HIV patients were retrospective evaluated, according to the Adult Treatment Panel III criteria for metabolic syndrome, Carr's clinical and biochemical criteria for LD, immunologic and ART status.

## Results

The prevalence of MB was 18,42% (14/76). LD was found on 57,89% (44/76) as atrophy (22%), hypertrophy (20%) or mixed (16%). Characteristics of the patients are: av. age=32 [22;59] years old , sex ratio M/F=31/45, smokers: 27/49, av. length of HIV diagnostic 5,52 [20;1] years, av. LCD4 nadir=264/mm3, av. LCD4 end-point=605,78/mm3, ART: 15 naïve, 19 first line INNRT regimen, 43 first line IP regimen. Neither LH nor LA are related to 3/5 criteria of MS: hyperglycemia (p<0,001; OR=54), hypertrigliceridemia (p=0,014; OR=6), arterial hypertension (p<0,001; OR=24,75). Male sex influences development of LA (p=0,01; OR=2,57) and MS (p=0,04; OR=3,27), while LH is more frequent on females (p=0,05; OR=5,83) . Only LA is influenced by young age (p=0,03), low LDC4 nadir (p<0,001) and PI —ART regimen (p=0,03). The length of HIV diagnostic is related either LA or LH, but not with MS. Over waist circumference was significantly associated to LH (p<0,001), but not to MS.

## Conclusions

1. The prevalence of LD is higher than MS on HIV adults.

2. Sex differences are recorded both for LD and MS.

3. HIV and ART status are mostly related to LA.

4. MS is an apart condition from LD on Romanian HIV infected adults.
